# Extra-Oral Taste Receptors—Function, Disease, and Perspectives

**DOI:** 10.3389/fnut.2022.881177

**Published:** 2022-04-04

**Authors:** Maik Behrens, Tatjana Lang

**Affiliations:** Leibniz Institute of Food Systems Biology at the Technical University of Munich, Freising, Germany

**Keywords:** taste receptors, gastrointestinal tract, pathogen defense, nutrient sensing, metabolism and endocrinology

## Abstract

Taste perception is crucial for the critical evaluation of food constituents in human and other vertebrates. The five basic taste qualities salty, sour, sweet, umami (in humans mainly the taste of L-glutamic acid) and bitter provide important information on the energy content, the concentration of electrolytes and the presence of potentially harmful components in food items. Detection of the various taste stimuli is facilitated by specialized receptor proteins that are expressed in taste buds distributed on the tongue and the oral cavity. Whereas, salty and sour receptors represent ion channels, the receptors for sweet, umami and bitter belong to the G protein-coupled receptor superfamily. In particular, the G protein-coupled taste receptors have been located in a growing number of tissues outside the oral cavity, where they mediate important processes. This article will provide a brief introduction into the human taste perception, the corresponding receptive molecules and their signal transduction. Then, we will focus on taste receptors in the gastrointestinal tract, which participate in a variety of processes including the regulation of metabolic functions, hunger/satiety regulation as well as in digestion and pathogen defense reactions. These important non-gustatory functions suggest that complex selective forces have contributed to shape taste receptors during evolution.

## Introduction

The concerted action of vision, olfaction, mechanoreception, and gustation enables humans to differentiate nutritionally valuable food items from inedible or even potentially harmful ones. The final gatekeeper is our sense of taste, which provides a rapid analysis of the relevant food-borne chemicals in the oral cavity prior to ingestion. To facilitate the detection of nutritionally relevant molecules among countless food constituents, the oral cavity is equipped with sensors for the five basic taste qualities salty, sour, sweet, umami (in humans mainly the taste of L-glutamic acid) and bitter (1). The sensing of table salt helps to maintain our body's electrolyte balance, sourness hints at the presence of unripe or spoiled food, sweet and umami tastes assess the energy content of food, and bitter sensing helps to avoid potentially harmful compounds (1). While taste sensing is limited to the sensory cells in the oral cavity, the detection of tastants by taste receptors continues throughout the alimentary canal as well as in other non-gustatory tissues. Among the extra-oral tissues expressing taste receptors, the airways have received considerable attention, because numerous cell types have been shown to respond to stimulation with tastants and the activation of these cells results in a wide range of profound physiological responses. The stimulation of solitary chemosensory cells in the respiratory epithelium of rodents with bitter compounds leads to respiratory depression (2–4) and elicits the discharge of antimicrobial peptides (5), the contact of ciliated lung cells increases their beat frequency (6) and bitter compound treatment of airway smooth muscle cells induces relaxation (7) making bitter compounds a relevant target for the treatment of asthma. These effects have been associated with the expression of the corresponding taste receptors in the various cell types. Other tissues shown to express taste receptors are the reproductive tract (8), urethra (9), skin (10–12), brain (13), heart (14, 15), pancreas (16, 17) and blood cells (18–22). For the sake of space, we will focus in this mini-review on the gastrointestinal (GI) tract and refer the interested reader to a number of comprehensive recent review articles for further reading (23–25). Before discussing the function of taste receptors in the GI tract, we will briefly introduce the receptors, their signaling elements and cells in their “original” environment, the taste system.

### Taste Cells and Receptors

The cells devoted to detect food constituents occur combined into taste buds, which consist of about 100 cells, on the tongue, the soft palate and the throat (26). The taste information gathered in the oral cavity are transmitted to the brain, where complex percepts are formed and innate as well as learned behaviors are evoked that regulate food intake. On the molecular level, tastants interact with taste receptors expressed in the taste receptor cells of the taste buds. The ionic taste stimuli are detected by ion channels, with the proton-gating channel otop-1 serving as sour taste receptor (27–29) and the epithelial sodium channel ENaC acting as salt taste receptor (30, 31). While the identity of otop-1 is meanwhile firmly established, the exact composition of the salt sensor is still a matter of debate (32–34). The receptors for sweet, umami and bitter taste belong to the large superfamily of G protein-coupled receptors (GPCR). The 3 *TAS1R* (taste 1 receptor) genes code for heteromers that assemble the predominant umami taste receptor, TAS1R1/TAS1R3 (35, 36), the sweet taste receptor, TAS1R2/TAS1R3 (37–42), and exert long extracellular so-called venus flytrap domains typical for class C GPCRs. In contrast, the 25 putatively functional bitter taste receptors belong to the TAS2R (taste 2 receptor) gene family with short amino termini (43–45).

Upon activation of one of the taste-GPCRs a signaling cascade centered around the IP_3_/Ca^2+^ second messenger system is initiated [for a review see (46)]. Briefly, depending on whether a sweet, umami, or bitter taste receptor cell is activated, a heterotrimeric G protein complex is recruited and, after GDP to GTP exchange, dissociates into the α-subunit and the βγ-heterodimer. The βγ-subunits in turn activate phospholipase Cβ2 resulting in the generation of IP_3_ from the membrane-associated precursor PIP_2_. Subsequently, the release of calcium ions from the lumen of the endoplasmatic reticulum via the receptor IP_3_R3 is triggered. Next, the elevated level of cytosolic calcium ions opens the cation-channels TRPM4 and TRPM5, which allow the influx of sodium ions into the cell causing depolarization. Finally, voltage-gated sodium channels open and the neurotransmitter ATP is released through the voltage-gated channel calcium homeostasis modulator 1 and 3 complex resulting in the activation of puringergic afferent nerve fibers and signal propagation toward the central nervous system.

### Tastant Reception in the GI Tract

Apart from its role in nutrient absorption, the GI tract is a site where nutrient sensing occurs and complex biological responses involving humoral and neural signals are triggered. The first hints that some of these responses may involve components of the taste transduction system came from the detection of α-gustducin, a Gα-subunit first identified in the rodent gustatory system (47), in brush cells of the stomach and small intestine (48). Nowadays, sweet, umami and bitter taste receptors and all canonical taste signaling components have been detected in GI tissues from stomach to colon (23–25). Moreover, a variety of GI cell types expressing the taste signaling molecules including enteroendocrine cells, brush cells, goblet cells, and Paneth cells have been discovered and nutrient sensing mechanisms involving taste-like signaling molecules were proposed (23–25).

### Sweet, Umami and Bitter Sensing in the GI Tract

Already the observation that the taste-related signaling molecule α-gustducin is expressed in brush cells of the GI tract raised the question if also other components involved in taste sensation might play a role in nutrient sensing in the alimentary canal. Indeed, over the past two decades all canonical taste signaling elements including the G protein-coupled receptors for sweet, umami and bitter detection have been identified in the GI tract of vertebrates. Moreover, a number of physiological functions of taste GPCR-mediated signaling have emerged. Here, we will point out only some key aspects of GI taste signaling, the interested reader is referred to one of the recent full-length review articles (24, 25).

#### Taste Receptor-Expressing Cell Types

**Figure 1:**
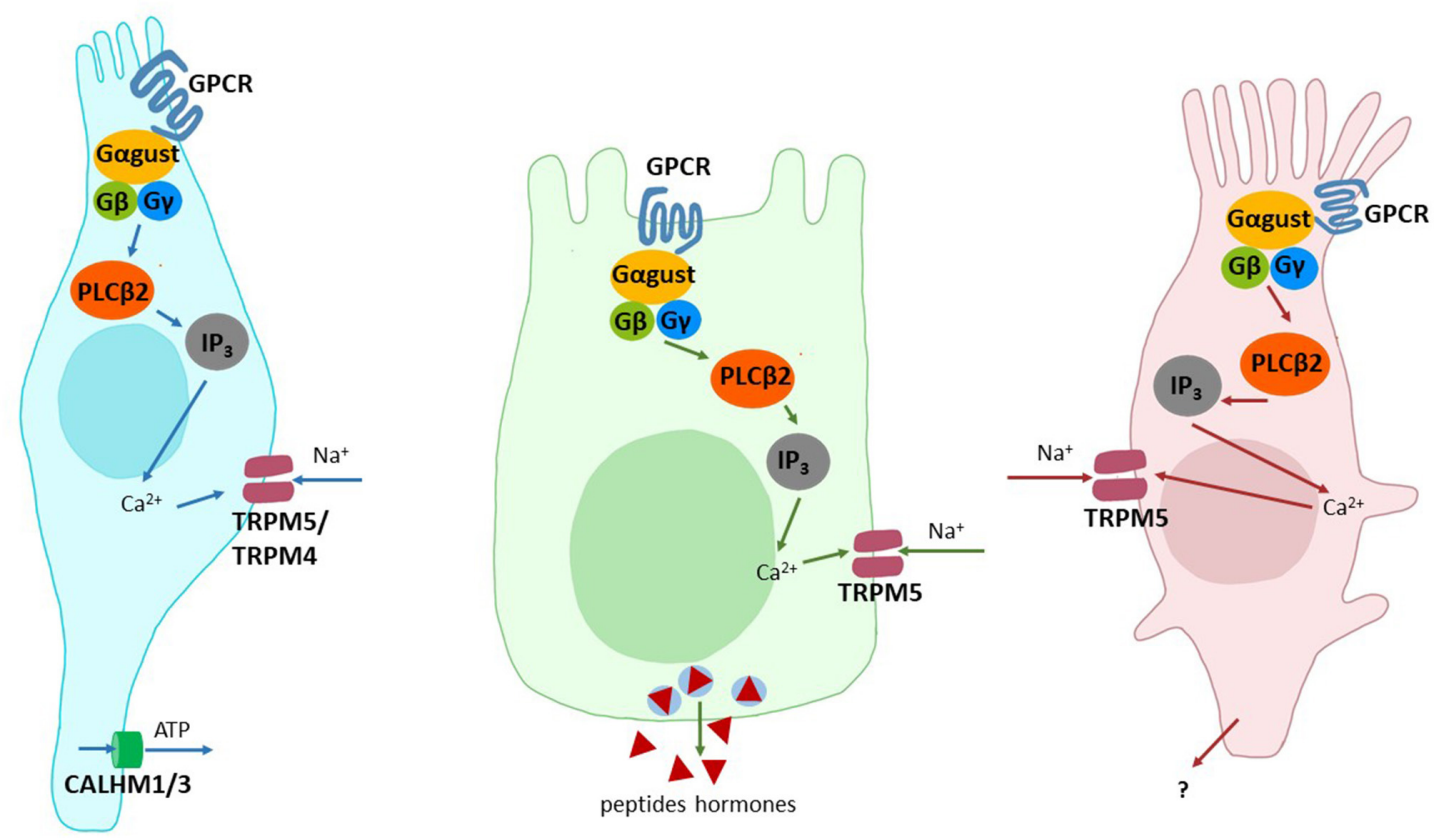
Cell types involved in the detection of taste stimuli. Shown are schematic drawings of type II taste receptor cells (left), enteroendocrine cells of the GI tract (middle) and brush cells (right). The signal components involved/implied in signal transduction are depicted: Adenosine triphosphate (ATP), calcium ions (Ca^2+^), Calcium homeostasis modulator 1 and 3 (CALHM1/3), G protein-coupled receptor (GPCR), G protein alpha subunit α-gustducin (Gαgust), G protein beta subunit (Gβ), G protein gamma subunit (Gγ), Inositol 1,4,5-trisphosphate (IP_3_), Phospholipase C beta 2 (PLCβ2), sodium ions (Na^+^), transient receptor potential cation channel subfamily M members 4 and 5 (TRPM5/4).

Although a larger number of cell types have been implicated in taste receptor-mediated signaling, two types of cells stand out because of their central role(s) and frequent implications in taste-related signaling events (Figure 1). The first cell type are the brush cells, which are frequently also named tuft cells or solitary chemosensory cells. These cells occur throughout the alimentary tract as individual cells, which are equipped with an apical tuft of microvilli [for a review see (50)]. They were shown to express sweet, umami and bitter taste receptors [but cf. (51)] as well as canonical taste signaling elements such as α-gustducin, PLCβ2, TRPM5 (52). It is believed that brush cells are capable to signal their activation via the transmitter acetylcholine in a paracrine fashion (53). The second cell type are so-called enteroendocrine cells, which can be further subdivided depending on the peptide hormones they secrete upon activation. Also the enteroendocrine cells were identified to express the already mentioned taste-GPCRs for sweet, umami and bitter sensing and the canonical taste-signaling elements. Stimulation of enteroendocrine cells results in the release of important peptide hormones involved in metabolic regulation, such as GLP-1 from enteroendocrine L cells, GIP from K cells, hunger-satiety regulation, such as ghrelin from P or X cells to name just a few [for a review see (54)]. Other, less well investigated cell types implicated in tastant-induced signaling in the GI tract include enterocytes (55), Paneth (56) and goblet cells (20). It is important to emphasize that the expression of sweet, umami and bitter taste receptors in a given cell type does not imply that individual cells actually house all three taste receptor types. While some cell lines of enteroendocrine origin may indeed contain taste receptors for multiple taste modalities, *in vivo* data on the co-expression does not exist to date.

#### Tastant-Induced Functions in the GI Tract

Quite a number of physiological roles have been assigned to taste receptor-mediated signaling in the GI tract. However, not all physiological responses triggered by tastants must occur via the activation of taste receptors, in fact, in many cases where taste receptors are implicated in GI tastant sensing additional research is warranted. Among the best investigated processes elicited by tastants in the GI tract are peptide hormone secretions from various enteroendocrine cell types. One of these hormones is the incretin hormone GLP-1 (glucagon-like peptide-1) which is produced by enteroendocrine L cells. The cells express both TAS1R subunits, namely TAS1R2 and TAS1R3, constituting the functional sweet taste receptor as well as the canonical taste signaling elements. Challenging the cells with sweet compounds results in the acute release of GLP-1 and the subsequent insulin secretion from pancreatic beta-cells leading to a reduction of blood sugar levels and, in case of chronic stimulation, an elevated absorptive capacity of intestinal enterocytes via the upregulation of the transport molecule SGLT-1 (57, 58). Also bitter compounds, such as KDT-501, a synthetic derivative of hop bitter compounds, have been shown to result in elevated GLP-1 levels in the blood of mice (59). Whether this implies co-expression of sweet and bitter taste receptors in L cells or suggests the existence of specialized subpopulations of sweet and bitter responsive L cells is unknown. Further effects of GI bitter stimulation on hormone secretions are the release of CCK (cholecystokinin) from I cells and a subsequent delayed gastric emptying and conditioned taste aversion (60, 61). Interestingly, bitter tastant-responsive X/A-like cells in the stomach also facilitate the release of the hunger-inducing peptide hormone ghrelin (62), a fact which appears counterintuitive on the first glance with the above reported CCK-effects. Not all presumably taste receptor-mediated GI effects involve necessary hormonal signaling events. It was shown that the bitter compound caffeine regulates via bitter taste receptors acid secretion in the human stomach (63) and in the colon of rodents an increased fluid secretion into the lumen (64). Moreover, the modulation of the intestinal motility via the interaction of selective bitter substances with bitter taste receptors in intestinal smooth muscle cells has been reported (65).

**Figure 2:**
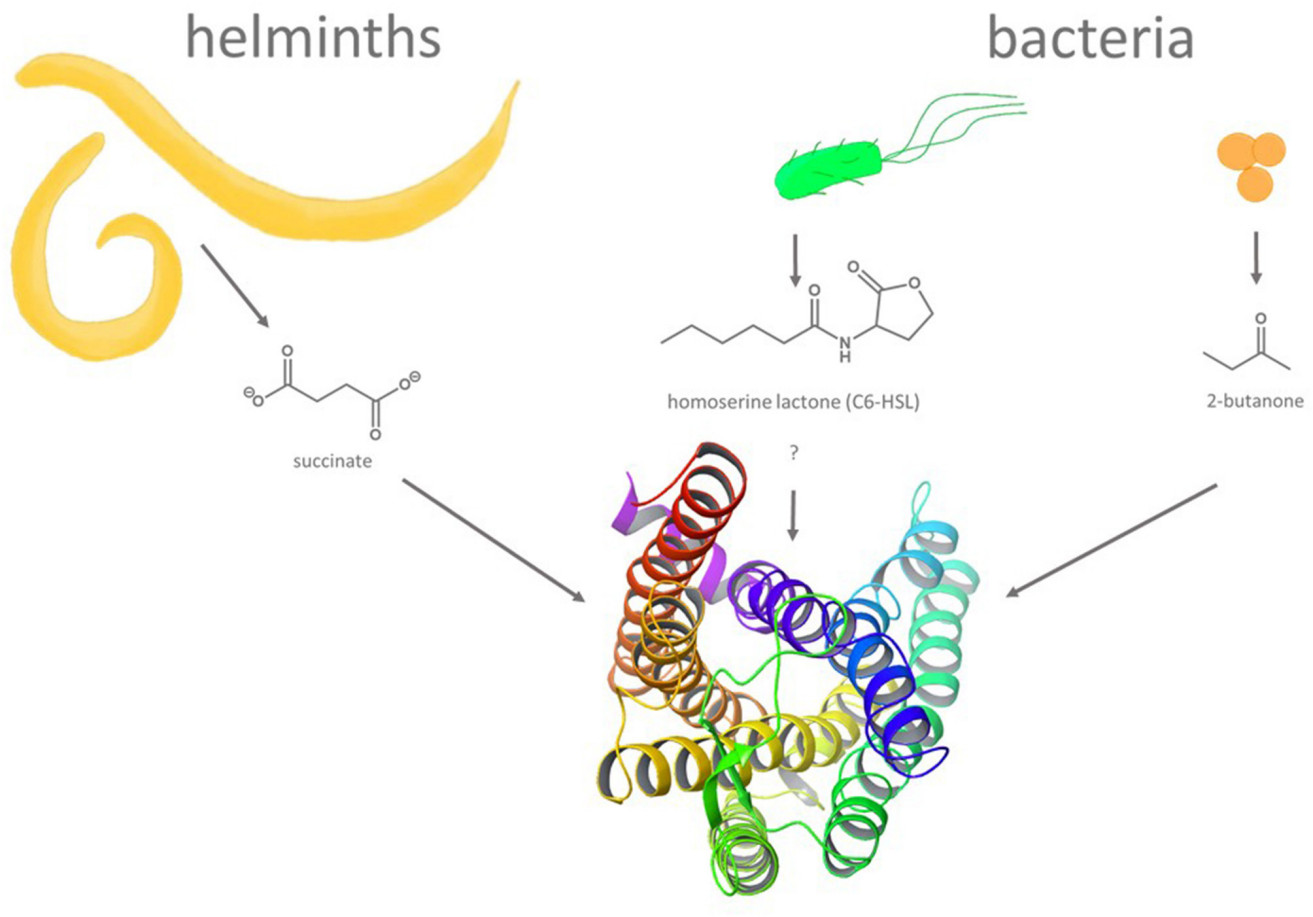
Molecules and organisms implicated in triggering GPCR-mediated defense reactions in non-gustatory epithelia. Gastrointestinal infections with succinate secreting helminths and bacteria secreting quorum-sensing molecules (homoserine lactones, e.g., C6-HSL) or metabolites (e.g., 2-butanone) are believed to result in the activation of GPCRs including bitter taste receptors. A ribbon structure (seen from the top) of a homology model of human bitter taste receptor TAS2R14 [derived from (49), modified with Maestro 12.9 software (Schrodinger)], one of the TAS2Rs implicated in innate immune responses, is depicted at the bottom.

Compared to enteroendocrine cells, intestinal brush cells seems to play a very different role, the defense of pathogenic organisms (66–68) (Figure 2). In fact, even though brush cells have been demonstrated to express all elements required for taste-GPCR signal transduction, albeit with some deviations from the canonical type II taste cell pathway (52), the taste receptors themselves were not detected in all studies [cf. (51)]. Nevertheless, brush cells respond to helminth and protist infections as well as to bacterial dysbiosis [for a review see (69)] and may indeed rely on the activation of bitter taste receptors (70). A central role in the pathogen response against these intestinal intruders was demonstrated for SUCNR1 (71, 72), a succinate-sensing GPCR (also known as GPR91) (73). Indeed, succinate is released by various pathogens [e.g., protozoa (72)] triggering IL-25 discharge from brush cells (72) in an α-gustducin- and TRPM5-dependent fashion (71) and a subsequent activation of group 2 innate lymphoid cells to promote pathogen removal (74). As succinic acid has been associated with a umami-like orosensory perception (75, 76), this process could be judged to represent an activity by a tastant-like substance. Although the majority of tastant or tastant-like molecules triggering important physiological responses in the GI tract have been associated with enteroendocrine or brush cells, other bitter taste receptor expressing cell types such as Paneth cells (56) or goblet cells (20) were shown recently to play critical roles in innate immune responses as well (77).

## Discussion

After the discovery of taste receptors outside the oral cavity, the research field of extra-oral taste receptors practically exploded. Taste receptors were found in an increasing number of tissues and were associated with numerous roles. Whereas, some of the most optimistic appraisals had to be corrected, other proposed opportunities solidified over the years resulting in realistic research goals such as the use of bitter compounds as asthma medication or compounds to improve metabolic functions.

One research gap that exists since the discovery of non-gustatory taste receptors and the subsequent investigations of their physiological roles is the firm association of taste stimuli with specific cellular functions and the unambiguous involvement of the corresponding taste receptors in this process. Moreover, many observations of physiological effects in tastant-responsive GI cells were rather broadly assigned to specific cell types, which may underestimate diversity with regard to taste receptor expression and function. Another gap comes from the observation that a large number of animals exhibit taste receptor pseudogenizations, which usually correlates well with their nutrition, however, it has so far not been investigated how these animals compensate for the loss of those receptors and their function in extra-oral tissues.

In summary, research on taste receptor functions outside the gustatory system has become a topic of great interest with future prospects ranging from more healthy nutrition to even using tastants and/or their derivatives for medicinal treatments.

## Author Contributions

The manuscript was written together by MB and TL. All authors contributed to the article and approved the submitted version.

## Funding

The authors declare that this study received funding from Soremartec Italia S.R.L. The funder was not involved in the study design, collection, analysis, interpretation of data, the writing of this article or the decision to submit it for publication.

## Conflict of Interest

The authors declare that the research was conducted in the absence of any commercial or financial relationships that could be construed as a potential conflict of interest.

## Publisher's Note

All claims expressed in this article are solely those of the authors and do not necessarily represent those of their affiliated organizations, or those of the publisher, the editors and the reviewers. Any product that may be evaluated in this article, or claim that may be made by its manufacturer, is not guaranteed or endorsed by the publisher.
